# ﻿*Camelliazijinica* (Theaceae), a new species endemic to Danxia landscape from Guangdong Province, China

**DOI:** 10.3897/phytokeys.237.114768

**Published:** 2024-01-31

**Authors:** Min Lin, Qin-Liang Ye, Zhi-Jian Zhang, Wen-Bo Liao, Qiang Fan

**Affiliations:** 1 State Key Laboratory of Biocontrol and Guangdong Provincial Key Laboratory of Plant Resources, School of Life Sciences, Sun Yat-sen University, Guangzhou 510275, China Sun Yat-sen University Guangzhou China; 2 Zijin Baixi Provincial Nature Reserve of Guangdong, Heyuan 517400, China Zijin Baixi Provincial Nature Reserve Heyuan China

**Keywords:** *Camellia*, Danxia landscape, morphology, new species, phylogeny

## Abstract

A new species of the genus *Camellia* (Theaceae), *Camelliazijinica*, discovered in the Danxia landscape from Guangdong Province, China, is characterized and illustrated. Phylogenetic analysis based on chloroplast genomes suggested its affinity with *C.drupifera*, *C.oleifera* and *C.fluviatilis*, however, it morphologically differs from all of the latter by leaf shape and size. Phonologically, it most closely resembles *C.microphylla*, but can be distinguished from the latter by its young branchlets glabrous (vs. densely pubescent), fewer bracteoles and sepals, diverse leaf shape, midvein raised slightly with sparsely pubescent or glabrous (vs. prominently with densely pubescent) and leaf adaxially matt (vs. vernicose) when dried. By morphological and molecular analyses, *Camelliazijinica* represented a distinct new species of C.sect.Paracamellia.

## ﻿Introduction

*Camellia* L. is the largest genus in Theaceae, widely distributed across eastern and southern Asia (Chang 1981; Ming et al. 2000). More than twenty new *Camellia* species have been predominantly reported and described in China and Vietnam in recent years (Shi et al. 2018; Hu et al. 2019; Le et al. 2020; Nguyen et al. 2020; Xu et al. 2020; Yu et al. 2021; Ly et al. 2022; Quach et al. 2022; Ye et al. 2022). Moreover, China has the highest richness of *Camellia* species with over 80% of *Camellia* species, mainly distributed in Yunnan Province, Guangdong Province, Guangxi Province and Sichuan Province (Chang and Ren 1998; Ming and Bartholomew 2007). Among them, more than 60% of *Camellia* species were endemic to China (Ming and Bartholomew 2007).

The sect. Paracamellia was initially constructed by Sealy, and the sect. Oleifera was proposed by Chang (1981). Chang and Ren (1998) and Ming (1999) both observed morphological similarities between sect. Paracamellia and sect. Oleifera, and Ming merged sect. Oleifera into sect. Paracamellia. However, Chang and Ren (1998) highlighted certain distinguishing features of sect. Paracamellia, such as smaller flowers, tinier fruits, shorter styles, shorter stamens, and thinner trunks compared to sect. Oleifera. In recent years, based on molecular analyses represented sect. Paracamellia was not monophyletic and nested with taxa of several sections, such as sect. Camellia, sect. Oleifera and sect. Archecamellia, etc., which the phylogenetic relationship of sect. Paracamellia and relatives need to be further explored and reconstructed through integrated means (Pang et al. 2022; Wu et al. 2022; Zan et al. 2023; Zhao et al. 2023).

During our field investigation of Guangdong Province in 2018, we noticed an unknown *Camellia* species, which can be easily misidentified as *C.microphylla* (Merr.) Chien. After several years of field observations and a precise comparison of relevant herbarium specimens, we confirmed it as a new member of the Camelliasect.Paracamellia based on morphological characteristics and molecular traits. In this study, we described and illustrated this new species.

## ﻿Materials and methods

### ﻿Morphological study

Field observations and collections of the new species were carried out from 2018 to 2023 in Zijin County, Guangdong Province, China. Morphological comparisons of the putative new species with the related species based on living plants, relevant literature and herbarium specimens, including “Flora Reipublicae Popularis Sinicae” (Chang and Ren 1998), “A Taxonomy of the Genus Camellia” (Chang 1981), “Flora of China” (Ming and Bartholomew 2007), the Chinese Virtual Herbarium (https://www.cvh.ac.cn/) and other recently described species and infraspecies of C.sect.Paracamellia (Ma et al. 2012; Lee and Yang 2019). Then we used nine morphological characters to distinguish each other. All the characters were measured and described by dissecting microscopes.

### ﻿Molecular analysis

Fresh leaf materials of individuals were collected and stored in silica gel for subsequent molecular experiments. Whole genomic DNA for each sample was extracted using the modified CTAB method (Doyle and Doyle 1987) and then purified using magnetic beads. A library was constructed for each sample by TruePrep DNA Library Prep Kit, which was then sent for Illumina sequencing on the Novaseq 6000 platform under standard operation procedure. Raw sequencing data were filtered with fastp v0.23.4 (Chen et al. 2018) to obtain clean data. Chloroplast genomes were assembled by GetOrganelle v1.7.7.0 (Jin et al. 2020) and annotated with cpGAVAS (Liu et al. 2012). The complete chloroplast sequences of *C.zijinica* were submitted to NCBI (https://www.ncbi.nlm.nih.gov/) and deposited with the accession number OR567094, OR567095, OR567096. Additionally, we extracted, sequenced, filtered, annotated the total DNA of *C.microphylla* with the accession number OR567093. Voucher specimens were deposited in the herbarium of Sun Yat-sen University (**SYS**).

We downloaded 50 accessions of chloroplast genomes containing 44 *Camellia* species and two related species as outgroups from the NCBI. The chloroplast genome sequences were aligned using MAFFT v7 (Katoh and Standley 2013). TrimAl v1.2 was applied to trim the alignment with the “gap out” model setting (Capella-Gutiérrez et al. 2009). Maximum likelihood (ML) and Bayesian inference (BI) were used to derive and construct their phylogenies respectively. ML tree was constructed using IQ-TREE v2.2.3 under 5,000 replicates of SH approximate likelihood ratio test (SH-aLRT) and 10,000 ultrafast bootstrap (UFBS) replicates (Nguyen et al. 2015). BI analysis was using MrBayes v.3.2.6 (Ronquist et al. 2012), and Markov chain Monte Carlo (MCMC) simulations were run for 1.6 million generations with one cold chain and three heated chains until the average standard deviation of split frequencies of these runs was <0.01 starting from random trees and sampling 1 of 100 generations, discarding the first 25% and the posterior probabilities were then estimated.

## ﻿Results

Based on morphological observations, *C.microphylla*, *C.brevistyla* (Hayata) Coh. St and *C.fluviatilis* Hand.-Mazz. exhibited morphological similarities with the new species. Among them, *C.microphylla* most resembles the new species, but *C.zijinica* has a more diverse leaf shape compared with *C.microphylla*, including elliptic, oblong-elliptic, obovate-elliptic or oblong-lanceolate, while *C.microphylla* displays oblong-elliptic or obovate-elliptic leaves, moreover, the leaf adaxially matt when dried (vs. vernicose). Differences between *C.zijinica* and *C.fluviatilis* are leaf shape and size, and the latter is lanceolate to narrowly lanceolate leaves, 5–9 × 1–1.5 cm (vs. 2–4.8 × 0.8–1.9 cm). Additionally, the leaf apex of *C.fluviatilis* is caudate-acuminate, which is a distinctive feature that sets it apart. *C.zijinica* and *C.brevistyla* differ not only in the shape and size of their leaves but also in having longer stamens and ovaries in the case of *C.brevistyla*. Furthermore, the young branchlets of *C.zijinica* are glabrous, while those of the other species are usually pubescent or glabrescent (Table 1).

**Table 1.** Morphological comparisons amongst *C.zijinica*, *C.brevistyla*, *C.microphylla* and *C.fluviatilis*.

Characters	*C.zijinica*	*C.brevistyla*	*C.microphylla*	*C.fluviatilis*
Leaf shape and size	elliptic, oblong-elliptic, obovate-elliptic or oblong-lanceolate , 2–4.8 × 0.8–1.9 cm	elliptic, obovate-elliptic, obovate-oblong or obovate, 3–5.5 × 1.5–3 cm	oblong-elliptic or obovate-elliptic , 2–3.5 × 1–1.3 cm	lanceolate to narrowly lanceolate, 5–9 × 1–1.5 cm
Leaf apices	rounded, acute or acuminate	acute	obtuse, rounded, acute	caudate-acuminate
Young branchlets	glabrous	pubescent to hirtellous	densely pubescent	puberulent, soon glabrescent
Petiole length	1–3 mm	3–5 mm	1–2 mm	2–5 mm
Bracteoles and sepals	4–6	7–8(-10)	6–7	8–9
Petals, shape and size	4–6, oblong-elliptic to obovate-elliptic, apex retuse, 0.7–1.1 × 0.4–0.5 cm	5–7, obovate to obovate-spatulate, apex retuse to deeply emarginate, 1–2.5(-3) × 0.4–1(-1.5) cm	5–7, broadly obovate, apex retuse, 0.8–1.1 × 0.5–0.8 cm	5–6, oblong-elliptic to oblanceolate, apex rounded to slightly retuse, 0.8–3 × 0.4–2 cm
Stamens	4–5 mm	5–10 mm	5–6 mm	5–7 mm
Capsule shape and size (diameter)	ovoid or subglobose, 1.2 cm	subglobose, 1.5–1.8 cm	ovoid, 1.5 cm	ovoid, 1.5–1.7 cm
Styles	3–4, 1–2 mm	3–4, 4–7 mm	3, 2–3mm	3, 4 mm

*Comparative data from: Chang and Ren (1998); Ming and Bartholomew (2007); Merrill (1927).

**Figure 1.**
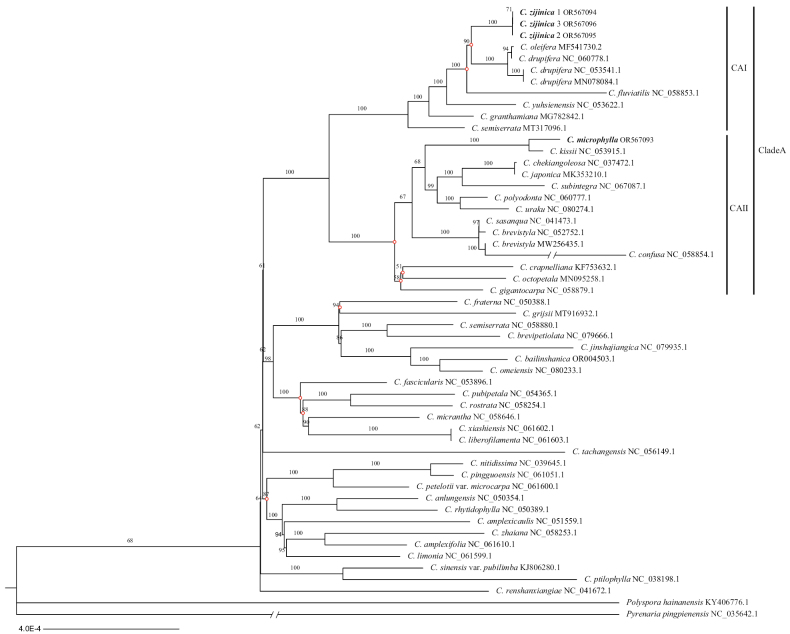
Maximum likelihood phylogenetic tree of *C.zijinica* and other 46 related species based on chloroplast genomes. Above the nodes of the tree, maximum likelihood ultrafast bootstrap support values were shown. The new species and *C.microphylla* were highlighted in bold. The red circles marked on the tree nodes indicated differences between the maximum likelihood and Bayesian inference. “CAI” and “CAII” refer to the two clades within CladeA.

Phylogenetic analysis by Maximum likelihood (ML) and Bayesian inference (BI) inferred from chloroplast genomes showed generally common results with highly supported values. The phylogenetic result using ML proved that this new species was sister to *C.drupifera*, *C.oleifera*, and successively grouped with *C.fluviatilis*, *C.yuhsienensis*, *C.granthamiana*, *C.semiserrata* in clade CAI with robust support values (BS = 100) (Fig. 1). However, the BI analysis presented a different arrangement in clade CAI, where the new species initially grouped with *C.fluviatilis* (PP = 0.90), and subsequently formed a clade with *C.drupifera*, *C.oleifera* (PP = 1.00) (Appendix 1). The species *C.microphylla* and *C.brevistyla* were nested within clade CAII, which was sister to clade CAI, together forming CladeA with highly supported values (PP = 1.00; BS = 100).

### ﻿Taxonomic treatment

#### ﻿ Camellia zijinica

Taxon classificationPlantaeEricalesTheaceae

M.Lin, Q.L.Ye & Q.Fan sp. nov.

8907A68B-C4B2-5CC7-BB55-3B7E1A7F852A

urn:lsid:ipni.org:names:77335468-1

**Figure 2.**
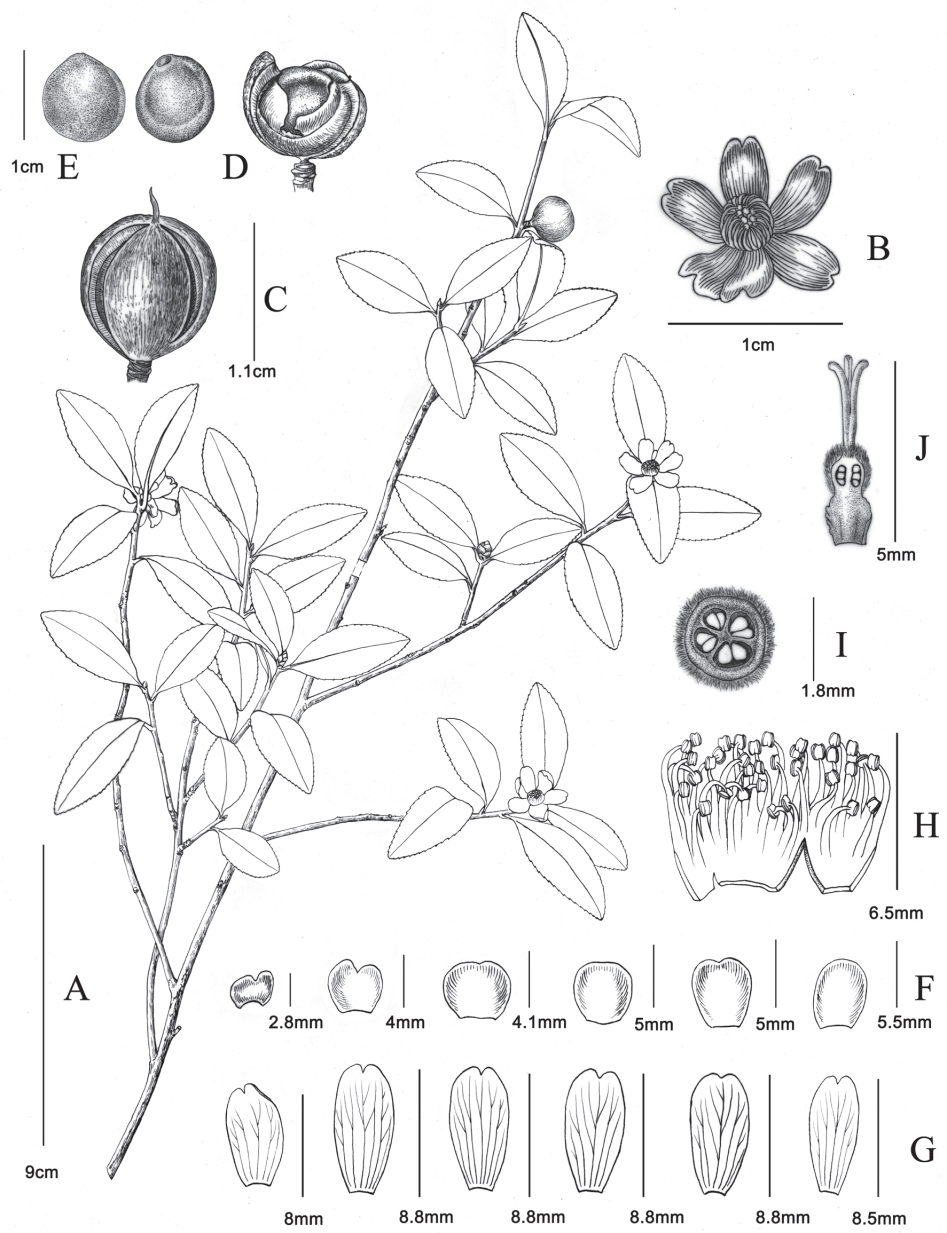
*Camelliazijinica* sp. nov. **A** flowering and fruiting branch **B** flower in front view **C** fully ripe fruit, tending to split **D** young fruit in longitudinal section **E** seeds in obverse and reverse sides **F** bracteoles and sepals **G** petals **H** stamens (incomplete) **I** ovary in transverse section **J** pistil in longitudinal section. Illustrated by Yun-Xiao Liu.

**Figure 3.**
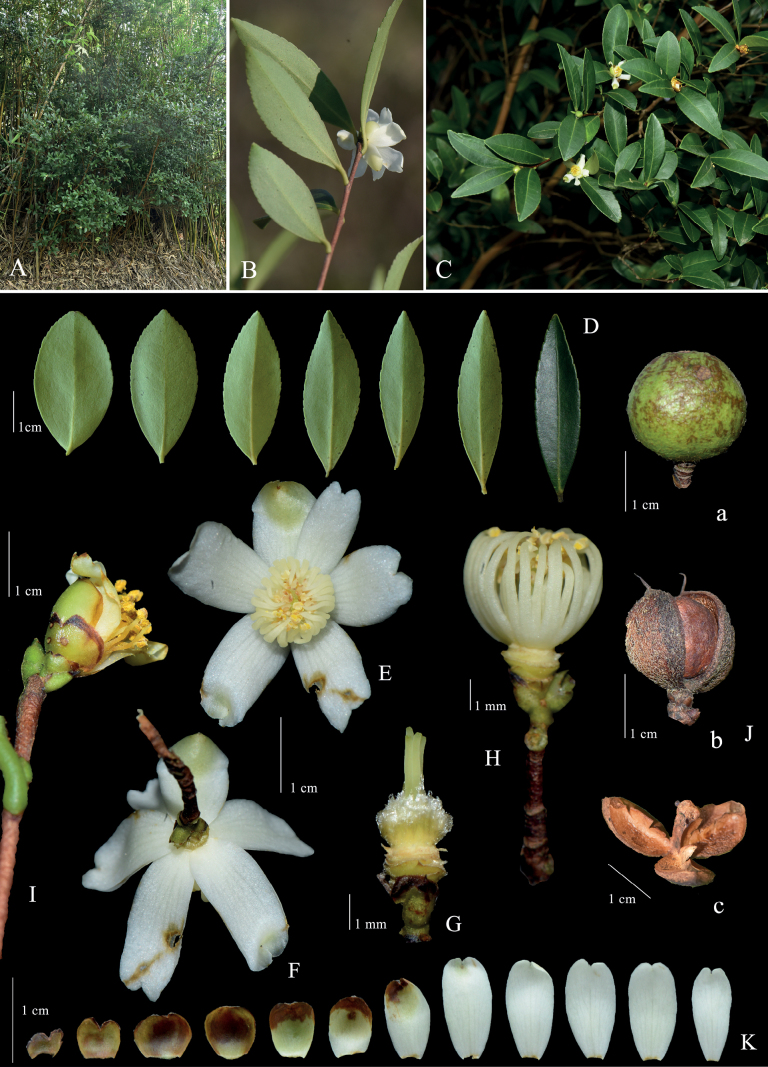
*Camelliazijinica* sp. nov. **A** habit **B, C** flowering branch **D** leaf shape **E** flower in front view **F** flower in back view **G** pistil and ovary **H** flowering branch, showing the stamens **I** flowering branch, showing the bracteoles and sepals **J** fruits, showing young to ripe (a-c) **K** bracteoles, sepals and petals. Photographed by Zhi-Ming Zhong, Qiang Fan and Min Lin.

Figs 2 , 3 Chinese name: 紫金短柱茶

##### Type.

China. Guangdong: Zijin County, Guzhu Town, Mount Yuewang, in mixed forests, 23°29'N, 114°44'E, 294 m a.s.l., 22 September 2018, *H.W. Wang* & *Q. Fan* 18084 (holotype: SYS00236945! isotypes: IBSC1010670! SYS00236946! SYS00236947! SYS00236948!)

##### Diagnosis.

*Camelliazijinica* is morphologically similar to *C.microphylla* in the flower shape and size, but differs from the latter by its young branchlets glabrous (vs. densely pubescent), the diverse leaf shape (elliptic, oblong-elliptic, obovate-elliptic or oblong-lanceolate vs. oblong-elliptic or obovate-elliptic), midvein raised slightly with sparsely pubescent or glabrous (vs. prominently with densely pubescent), fewer bracteoles and sepals (4–6 vs. 6–7), leaf adaxially matt when dried (vs. vernicose).

##### Description.

Small evergreen shrubs, 2–5 m tall; bark yellowish brown; young branchlets reddish brown, glabrous. ***Leaf*** blades elliptic, oblong-elliptic, obovate-elliptic or oblong-lanceolate, 2–4.8 × 0.8–1.9 cm, adaxially dark green, abaxially light green, thick papery to coriaceous; petiole 1–3 mm long, pubescent; midrib prominent on both surfaces, glabrous and sometimes adaxially sparsely pubescent, secondary veins 6–7 on each side, invisible; apex rounded, acute or acuminate; margin serrulate; base cuneate. ***Flowers*** solitary, terminal or axillary, 1.5–2.3 cm in diameter, subsessile. bracteoles and sepals 4–6, caducous, outside pubescent at base, inside glabrous, margin ciliate; outer bracteoles and sepals broadly semiorbicular, partial apex bifid; inner bracteoles and sepals suborbicular to oblong-elliptic. Petals 4–6, white, distinct, glabrous, oblong-elliptic to obovate-elliptic, apically 2-lobed, 7–11×4–5 mm. ***Stamens*** 33–40, 4–5 mm long, glabrous; outer filament whorl basally connate for 1–1.5 mm. ***Ovary*** 3–4-loculed, with 2 ovules per locule, about 2 mm in diameter, tomentose. ***Styles*** 3 or 4, 1–2 mm long, connate half from the base, apically 3–4-lobed. ***Capsule*** ovoid or subglobose, ca. 1.2 cm in diameter; pericarp 1–2 mm thick, splitting into 3–4 valves. ***Seeds*** semiglobose or globose, 9–10 mm in diameter, brown, glabrous.

##### Phenology.

Flowering from September to December, fruiting from January to September.

##### Etymology.

The specific epithet refers to Zijin County of Guangdong Province, the type locality of the new species.

##### Distribution and habitat.

Presently, *Camelliazijinica* is only known from its type locality, Zijin County of northeastern Guangdong. It is distributed in mixed forests of Danxia landscape at altitudes of 200–400 m a.s.l.

##### Conservation status.

Only two populations of a total of about 90 mature individuals were found currently. Thus, the species could be considered as Endangered (EN; D) status according to IUCN Red List criteria (IUCN Standards and Petitions Subcommittee 2022).

##### Additional specimens examined

**(paratypes).** China. Guangdong: Zijin County, Guzhu Town, near Mount Yuewang, 23°37'N, 115°10'E, 234 m a.s.l., 12 Oct. 2022 (fl. and fr.), *Z.M. Zhong 1012* (SYS); Zijin County, Guzhu Town, near Mount Yuewang, 23°29'N, 114°44'E, 310 m a.s.l., 7 June 2023 (young fr.), *Z.M. Zhong 0607* (SYS), the type locality, 21 July 2023 (young fr.), *Z.M. Zhong 0721* (SYS).

## ﻿Discussion

Geographically, *C.drupifera* and *C.oleifera* are distributed in southern China, belonging to sect. Oleifera. Although they share a similar distribution area with the new species, they exhibit distinct morphological characteristics, such as larger leaves, flowers and fruits compared to *C.zijinica*. Furthermore, *C.fluviatilis*, a sympatric species of the new species, shows similarities in gross morphology but can be easily distinguished by leaf shape and size. Instead, *C.microphylla* is distributed in a different distribution area from the new species, located in the Jiangxi, Guizhou, Zhejiang, Hunan and Anhui Provinces, China, but they most resembled in morphology. Traditionally, the identification of *C.microphylla* is associated with having the smallest leaves within the sect. Paracamellia (Chang and Ren 1998). Nevertheless, our careful comparisons revealed that its leaves were similarly small to those of *C.zijinica*.

The phylogenetic tree generated using ML and BI showed *C.zijinica* was grouped with *C.drupifera*, *C.oleifera* (BS = 90) and *C.fluviatilis* (PP = 0.90) with low support values, implying an unclear relationship among them. However, these species collectively exhibited the closest relationship to the new species. The species within CladeA belong to the sect. Oleifera, sect. Paracamellia, sect. Camellia, sect. Archecamellia, sect. Furfuracea, as classified in Flora Reipublicae Popularis Sinicae (Chang and Ren 1998). Additionally, they are classified in the sections of *Paracamellia*, *Camellia*, and *Heterogenea* based on Flora of China (Ming and Bartholomew 2007), suggesting a close relationship among these sections. Moreover, recent phylogenetic analyses of *Camellia* based on low-copy nuclear genes and chloroplast genomic regions have suggested that further investigation is needed to clarify the relationships among these *Camellia* sections (Zan et al. 2023; Wu et al. 2022). Nevertheless, our comprehensive comparisons and analyses revealed that *C.zijinica* was a distinct new species of sect. Paracamellia.

## Supplementary Material

XML Treatment for Camellia zijinica

## Figures and Tables

**Figure A1.**
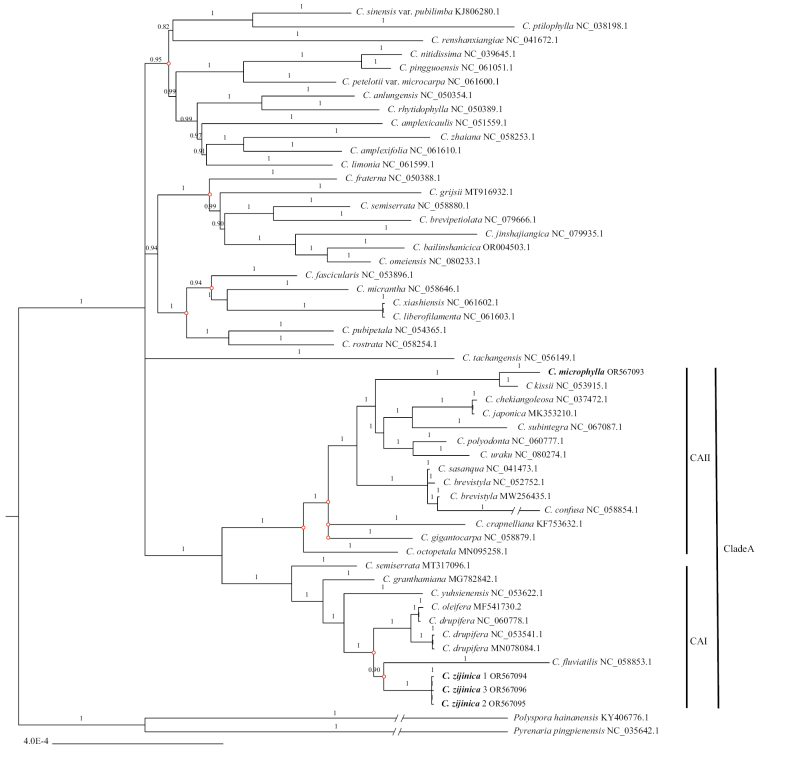
Bayesian inference phylogenetic tree of *C.zijinica* and other 46 related species based on chloroplast genomes. Above the nodes of the tree, Bayesian posterior probabilities support values were shown. The new species and *C.microphylla* were highlighted in bold. The red circles marked on the tree nodes indicate differences between the maximum likelihood and Bayesian inference. “CAI” and “CAII” refer to the two clades within CladeA.

## ﻿Additional information

### Author ORCIDs

Min Lin https://orcid.org/0000-0001-6342-1978

Wen-Bo Liao https://orcid.org/0000-0001-6839-9511

Qiang Fan https://orcid.org/0000-0003-4254-6936

### Data availability

All of the data that support the findings of this study are available in the main text.
